# Corn Protein-Derived Bioactive Peptides Protect Gastric Epithelial Cells from *Helicobacter pylori*-Induced Injury by Alleviating Oxidative Stress, Mitochondrial Dysfunction, and Inflammation

**DOI:** 10.3390/foods15152748

**Published:** 2026-08-05

**Authors:** Guanlong Li, Chenyang Ma, Zhengfei Miao, Yongchao Xie, Quanxin Wang, Xiaolan Liu, Xiqun Zheng

**Affiliations:** 1 Heilongjiang Provincial Key Laboratory of Corn Deep Processing Theory and Technology, College of Food and Bioengineering, Qiqihar University, Qiqihar 161006, China; 03580@qqhru.edu.cn (G.L.); 2025916387@qqhru.edu.cn (C.M.); 2021907279@qqhru.edu.cn (Z.M.); 2024916366@qqhru.edu.cn (Y.X.); wangquanxin979@163.com (Q.W.); 2 Engineering Research Center of Plant Food Processing Technology, Ministry of Education, Qiqihar 161006, China; 3 College of Food Science, Heilongjiang Bayi Agricultural University, Daqing 163319, China

**Keywords:** *Helicobacter pylori*, corn protein-derived bioactive peptides, GES-1 cells, oxidative stress, gastric diseases

## Abstract

*H. pylori* infection induces oxidative stress and inflammatory responses in gastric epithelial cells, which are key factors in the pathogenesis of gastritis and ulcers. Given the increasing threat of antibiotic resistance, non-antibiotic approaches that target host cell injury mechanisms are gaining considerable interest. While peptides derived from corn protein are known for their antioxidant and anti-inflammatory activities, whether they can alleviate *H. pylori*-induced gastric epithelial injury remains unclear. In this study, we evaluated the preventive effects of corn protein-derived bioactive peptides (CPDP-N), prepared by neutral protease hydrolysis, against *H. pylori*-triggered injury in human GES-1 cells. CPDP-N exhibited no cytotoxic effects, reduced *H. pylori*-induced intracellular ROS accumulation in a dose-dependent manner, and markedly increased the activities of intracellular antioxidant enzymes. Flow cytometry and fluorescence imaging demonstrated that CPDP-N attenuated the loss of mitochondrial membrane potential and relieved G0/G1 cell cycle arrest. CPDP-N markedly suppressed the secretion of the pro-inflammatory cytokines TNF-α, IL-1β, and IL-8, attenuated LDH release, and upregulated the anti-inflammatory cytokine IL-10. Moreover, CPDP-N markedly lowered the *H. pylori*-induced elevation of nuclear factor kappa-B (NF-κB) p65 protein, a key regulator of inflammatory signaling. These protective effects were accompanied by reduced intracellular ROS levels and lower NF-κB p65 abundance, suggesting the involvement of oxidative stress and NF-κB pathways. Collectively, these findings demonstrate that corn protein-derived peptides can protect gastric epithelial cells from *H. pylori*-induced oxidative and inflammatory injury, highlighting their potential as a dietary intervention for *H. pylori*-associated gastric diseases.

## 1. Introduction

*H. pylori* is a gastric pathogen that causes chronic gastritis and peptic ulcers and is strongly linked to the development of gastric cancer [1]. One of the most widespread chronic infections globally, *H. pylori* colonizes over half of the world’s population [2]. Although most infected individuals show no obvious symptoms, the infection initiates chronic gastric inflammation and may progress to severe gastric diseases [3]. Given its established pathogenicity, the International Agency for Research on Cancer (IARC) has classified *H. pylori* as a Group I carcinogen. Clinical guidelines in many countries recommend eradication therapy for all confirmed cases, irrespective of symptoms [4,5]. Over its long co-evolution with humans, *H. pylori* has become well adapted to the gastric environment, enabling it to evade immune clearance [6]. Standard treatment relies on a quadruple regimen that includes two antibiotics, a proton pump inhibitor, and bismuth [7]. Although effective, this approach is increasingly compromised by side effects and rising drug resistance. In most regions, resistance rates to clarithromycin and levofloxacin each exceed 15% [8]. For these reasons, the development of non-antibiotic strategies to combat *H. pylori* infection has become an urgent research priority.

*H. pylori*-induced gastric inflammation is a complex process driven by the interplay between host immune responses and bacterial virulence factors. Its cumulative effects contribute to the initiation and progression of various gastric diseases. Once *H. pylori* crosses the mucus layer, it binds to gastric epithelial cells through surface adhesins that engage host receptors. Its components, including urease, adhesins, and LPS, are then sensed by the innate immune system, which activates downstream inflammatory signaling. Once colonization is established, *H. pylori* releases virulence factors, including CagA and VacA, which directly damage gastric mucosal cells and exacerbate local inflammation by disrupting cell signaling and immune regulation [9,10,11]. Interrupting these pathogenic mechanisms through non-antibiotic approaches has therefore become a focus of current research aimed at preventing and alleviating *H. pylori*-associated gastric mucosal injury.

Our previous work has shown that corn protein-derived peptides produced by enzymatic hydrolysis can counteract *H. pylori* infection. In vitro assays demonstrated that the hydrolysate inhibited urease activity, suppressed bacterial adhesion, and scavenged free radicals [12]. However, whether these peptides can protect gastric epithelial cells from *H. pylori*-induced injury remains unclear. This study aimed to investigate the preventive effects of CPDP-N pretreatment on *H. pylori*-triggered oxidative stress and inflammatory responses in GES-1 cells and to explore the underlying mechanisms by measuring key cellular functional indicators and cytokine profiles.

## 2. Materials and Methods

### 2.1. Materials and Reagents

Corn gluten meal was supplied by Heilongjiang Longfeng Corn Development Co., Ltd. (Suihua, Heilongjiang, China). Neutral protease (24,000 U/mL) was sourced from Novo Nordisk (Bagsvaerd, Denmark). *H. pylori* strain 43504 was acquired from the American Type Culture Collection (ATCC, Manassas, VA, USA), and the GES-1 cell line was obtained from JianCheng Co., Ltd. (Nanjing, Jiangsu, China). The reactive oxygen species assay kit (S0033), cell cycle kit (C1052), and mitochondrial membrane potential kit (C2002) were purchased from Beyotime Biotechnology Co., Ltd. (Shanghai, China).

### 2.2. Production of Corn Protein-Derived Peptides via Neutral Protease Hydrolysis

According to our previous study, corn gluten meal was pretreated by extrusion and destarching [13]. A 15% (*w*/*v*, protein basis) suspension of the pretreated material was prepared in deionized water. Neutral protease was added at 400 U/g protein, and hydrolysis was performed at 45 °C and pH 7.0 for 120 min. After inactivating the enzyme by boiling for 15 min, the mixture was centrifuged at 4000 rpm for 10 min, and the resulting supernatant was collected and freeze-dried. The resulting corn protein-derived peptide mixture was designated CPDP-N.

A comprehensive characterization of CPDP-N, covering its amino acid profile, molecular weight distribution, peptide sequences, and antioxidant properties, was detailed in our earlier work [13]. In brief, peptides under 1 kDa made up 74.10% of the hydrolysate, with sequences identified via LC-MS/MS. CPDP-N also demonstrated strong DPPH and ABTS radical scavenging capacities.

### 2.3. Culture of Helicobacter pylori

*H. pylori* was cultured as described previously [13]. Briefly, before infection, the bacterial suspension was adjusted to an OD_600_ of approximately 0.1 (10^8^ CFU/mL).

### 2.4. GES-1 Cell Culture

GES-1 cells were cultured at 37 °C in a humidified atmosphere containing 5% CO_2_. Cell morphology and growth were monitored daily. At 80–90% confluence, cells were subcultured or cryopreserved as needed.

### 2.5. Effect of CPDP-N on GES-1 Cell Proliferation

GES-1 cells in the logarithmic growth phase were harvested and collected by centrifugation at 1000 rpm for 5 min. The cell pellet was resuspended in DMEM complete medium and adjusted to a density of 1 × 10^5^ cells/mL. A 100 μL aliquot was seeded into each well of a 96-well plate and incubated overnight. After cell attachment, 100 μL of CPDP-N solution (prepared in serum-free DMEM) was added to yield final concentrations of 25, 50, 62.5, 125, 250, and 500 μg/mL. The blank control group received 100 μL of serum-free DMEM, with eight replicate wells per group. Following 24 h of incubation, MTT solution was introduced at a final concentration of 0.5 mg/mL and incubated for another 4 h. The medium was then carefully removed, and 150 μL of DMSO was added to each well. The plate was shaken for 10 min to ensure complete dissolution, and the absorbance was recorded at 570 nm. Cell viability was calculated according to the following formula:

$$\mathrm{Cell}\;\mathrm{viability}\;\left(\%\right)=\;\frac{{\mathrm{OD}}_{\mathrm{sample}}}{{\mathrm{OD}}_{\mathrm{control}}}\;100$$

### 2.6. Measurement of Intracellular ROS in GES-1 Cells

Intracellular ROS levels were detected according to the protocol of Liu et al. [14]. GES-1 cells were plated in 96-well plates at 1 × 10^5^ cells/mL (100 μL/well) and incubated overnight. After cells had adhered, CPDP-N solutions (prepared in serum-free DMEM) were added to give final concentrations of 50, 100, and 200 μg/mL. Blank control and *H. pylori* infection model groups were treated with serum-free DMEM alone. Each group comprised eight replicate wells. After 24 h, the medium was aspirated, and all groups except the blank control were infected with *H. pylori* at an MOI of 100:1 for an additional 6 h. The supernatant was removed, and the wells were rinsed twice with PBS. Cells were then loaded with 100 μL of 10 μM DCFH-DA for 20 min. After the probe was removed, fluorescence images were acquired with a fluorescence microscope. Subsequently, cells were trypsinized and subjected to flow cytometry. Whole-well fluorescence intensity was quantified using a microplate reader at excitation/emission wavelengths of 485/530 nm, and the values were normalized to the control group (set as 100%).

### 2.7. Effect of CPDP-N on Cell Cycle in H. pylori-Infected GES-1 Cells

Cell cycle distribution was evaluated according to Zhang et al. [15]. GES-1 cells were seeded into six-well plates at 1 × 10^5^ cells/mL (2 mL/well) and cultured overnight. After cell attachment, 500 μL of CPDP-N solutions (prepared in serum-free DMEM) were added to give final concentrations of 50, 100, and 200 μg/mL. Blank control and *H. pylori* infection model groups received 500 μL of serum-free DMEM alone. Following 24 h of incubation, the medium was discarded, and all groups except the blank control were exposed to 500 μL of *H. pylori* suspension at an MOI of 100:1 for an additional 6 h. The supernatant was subsequently removed, and the cells were washed twice with PBS, trypsinized, and collected. Cell cycle distribution was assessed by flow cytometry using a commercial kit (Beyotime) according to the manufacturer’s instructions.

### 2.8. Measurement of Mitochondrial Membrane Potential in H. pylori-Infected GES-1 Cells

GES-1 cells were seeded, treated with CPDP-N, and infected with *H. pylori* as described in Section 2.7. After removing the supernatant, cells were washed twice with PBS, harvested by trypsinization, and stained with a mitochondrial membrane potential assay kit following the manufacturer’s protocol. The membrane potential was then assessed by flow cytometry.

### 2.9. Assessment of Apoptosis-Related Gene Expression in H. pylori-Infected GES-1 Cells

The effect of CPDP-N on apoptosis-related gene expression was evaluated according to Kim et al. [16]. GES-1 cells were seeded, treated with CPDP-N, and infected with *H. pylori* as described in Section 2.7. Following infection, total RNA was isolated with the Omega Tissue RNA Kit (Omega, Norcross, GA, USA) and reverse-transcribed into cDNA using the TaKaRa PrimeScript™ IV Reverse Transcription Kit (TaKaRa, Beijing, China). qRT-PCR was conducted on a QuantStudio system with ThermoFisher SYBR Green Master Mix (ThermoFisher, Waltham, MA, USA), and *GAPDH* was used as the internal reference. Primer sequences are shown in Table 1.

### 2.10. Effects of CPDP-N on Antioxidant Enzyme Activities and Inflammatory Cytokine Levels in H. pylori-Infected GES-1 Cells

GES-1 cells were seeded, treated with CPDP-N, and infected with *H. pylori* as described in Section 2.7. Following infection, cells were washed twice with PBS, trypsinized, and collected into 2 mL tubes. After homogenization on ice and centrifugation at 10,000 rpm for 10 min, the supernatant was stored at −80 °C until analysis. The activities of SOD, GSH-Px, and CAT in cell lysates were measured using commercial assay kits according to the manufacturer’s instructions. The levels of IL-8, IL-1β, TNF-α, LDH, IL-10, and NF-κB p65 in the supernatant were measured by ELISA.

### 2.11. Statistical Analysis

**Figure 1:**
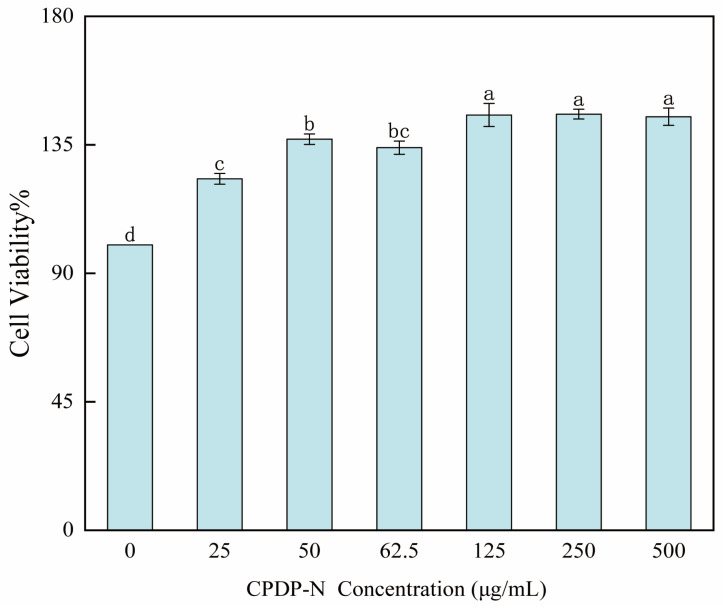
Effect of CPDP-N on cell proliferation. Data are presented as mean ± SD (*n* = 8 replicate wells). Different lowercase letters represent significant differences between groups (*p* < 0.05).

Statistical analyses were carried out using IBM SPSS Statistics 26, with results expressed as mean ± standard deviation (SD). Differences among groups were evaluated by one-way ANOVA followed by Tukey’s HSD post hoc test. All figures were generated with Origin 2019 software. The MTT assay (Figure 1) was performed with eight replicate wells per group (*n* = 8). All other experiments (ROS, cell cycle, mitochondrial membrane potential, qRT-PCR, and ELISA) were conducted in three independent biological replicates (*n* = 3).

## 3. Results and Discussion

### 3.1. Effect of CPDP-N on GES-1 Cell Proliferation

Cell proliferation rate is a key indicator for evaluating sample safety. This section aimed to investigate the preventive effect of CPDP-N against *H. pylori*-induced injury in GES-1 cells. It was therefore essential to first assess whether CPDP-N exerts any cytotoxic effect on these cells. GES-1 cells are human gastric mucosal epithelial cells originally isolated from the gastric epithelium of a 9-month-old fetus and immortalized by SV40 virus transfection. They serve as an important model for studying human gastric mucosal injury and tumorigenesis. Because *H. pylori* primarily adheres to and colonizes the human gastric mucosa, GES-1 cells were selected for this study.

Figure 1 illustrates that within the concentration range of 0–500 μg/mL, cell viability in all CPDP-N-treated groups was consistently higher than that of the control (*p* < 0.05), indicating that CPDP-N exhibited no detectable cytotoxicity in GES-1 cells. Compared with the control, viability increased in a concentration-dependent manner and remained significantly elevated at all doses (*p* < 0.05). The viability values exceeding 100% of the control are likely attributable to the nutritional contribution of CPDP-N. Peptides below 1 kDa accounted for 74.10% of the hydrolysate [13] and may be readily taken up and utilized by cells. Because the MTT assay was performed in serum-free medium, the amino acids and small peptides in CPDP-N could have served as metabolic substrates under these nutrient-limited conditions, supporting proliferation beyond the basal level. Within the concentration range tested, CPDP-N treatment for 24 h showed no toxic or adverse effects on GES-1 cells, supporting its safety as a functional food ingredient.

### 3.2. CPDP-N Significantly Attenuated H. pylori-Induced Intracellular ROS Accumulation in GES-1 Cells

Reactive oxygen species (ROS) are molecules generated from the partial reduction in intracellular oxygen, including hydroxyl radicals and superoxide anions [17]. ROS can bind to and damage bioactive macromolecules, thereby impairing cellular function and causing oxidative injury. Intracellular ROS levels are therefore positively correlated with the extent of oxidative damage. In this assay, DCFH-DA readily enters cells and, upon oxidation by intracellular ROS, is converted to fluorescent DCF. Thus, the fluorescence intensity serves as an indirect measure of intracellular ROS levels.

**Figure 2:**
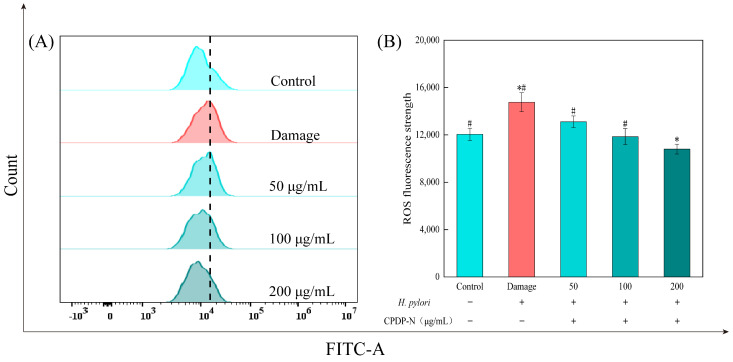
CPDP-N attenuates intracellular ROS accumulation in GES-1 cells. (**A**) Flow cytometry histograms; (**B**) Fluorescence intensity quantified by flow cytometry. * *p* < 0.05 vs. control; ^#^ *p* < 0.05 vs. *H. pylori*-infected group. In the figure, “+” indicates the addition of the corresponding substance, and “−” indicates that it has not been added. Data are presented as mean ± SD (*n* = 3).

As shown in Figure 2, in comparison to the control, the *H. pylori*-infected group exhibited a pronounced elevation in intracellular ROS levels (*p* < 0.05), indicative of severe oxidative stress and substantial ROS accumulation. CPDP-N pretreatment reduced ROS in a dose-dependent manner, and at concentrations of 50 μg/mL and above, ROS levels no longer differed significantly from those of the control (*p* > 0.05). These findings confirm that CPDP-N pretreatment effectively mitigated *H. pylori*-induced oxidative stress and decreased intracellular ROS generation.

**Figure 3:**
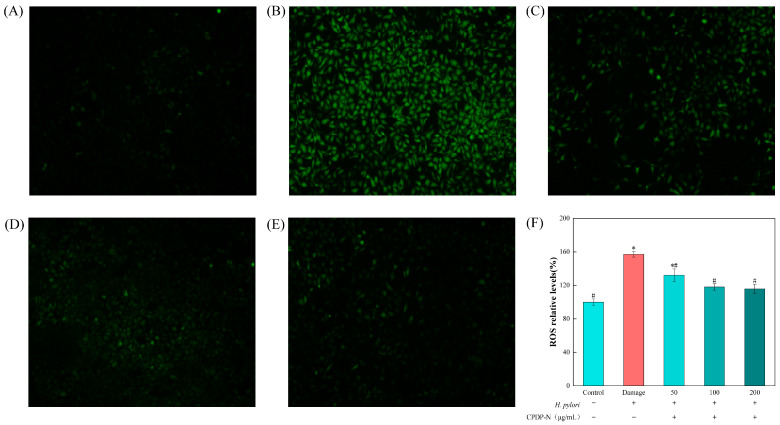
Fluorescence microscopy images of GES-1 cells. Green fluorescence corresponds to DCF; a stronger green signal indicates higher fluorescence intensity and thus elevated intracellular ROS levels. (**A**) Blank control; (**B**) *H. pylori*-infected damage group; (**C**) 50 μg/mL CPDP-N; (**D**) 100 μg/mL CPDP-N; (**E**) 200 μg/mL CPDP-N. (**F**) Quantification of whole-well fluorescence intensity determined by a microplate reader. * *p* < 0.05 vs. control; # *p* < 0.05 vs. *H. pylori*-infected group. In the figure, “+” indicates the addition of the corresponding substance, and “−” indicates that it has not been added. Data are expressed as relative fluorescence intensity (% of control) and presented as mean ± SD (*n* = 3).

Figure 3 shows representative fluorescence microscopy images of GES-1 cells. The green fluorescence corresponds to DCF, and a stronger green signal reflects higher fluorescence intensity and thus elevated intracellular ROS levels. After CPDP-N pretreatment (C–E), the intracellular fluorescence intensity decreased in a concentration-dependent manner, indicating that CPDP-N effectively reduced intracellular ROS and alleviated oxidative stress. Quantitative analysis of whole-well fluorescence intensity (Figure 3F) confirmed these observations. Taking the control as 100%, the *H. pylori*-infected group showed a marked increase in fluorescence intensity (*p* < 0.05). At 200 μg/mL, the relative intensity was comparable to that of the control, in agreement with the flow cytometry results presented in Figure 2.

This effect may be closely associated with the potent antioxidant activity of CPDP-N. Wang et al. reported that corn peptide pretreatment improved the viability of H_2_O_2_-injured Caco-2 cells by markedly enhancing the activities of antioxidant enzymes, including glutathione reductase, superoxide dismutase, and γ-glutamylcysteine synthetase, thereby mitigating oxidative damage [18]. Yu et al. found that the corn pentapeptide YFCLT alleviated oxidative injury in HepG2 cells by increasing intracellular antioxidant enzyme activities and consequently decreasing ROS levels [19]. Collectively, these findings suggest that CPDP-N may protect GES-1 cells against *H. pylori*-induced oxidative damage by upregulating antioxidant enzyme activities and thereby accelerating ROS clearance. Furthermore, the anti-adhesive effect of CPDP-N on *H. pylori*, which reduces bacterial colonization on the surface of GES-1 cells, may also contribute to the attenuation of oxidative injury [12]. Therefore, the changes in intracellular antioxidant enzyme activities were further examined.

### 3.3. Effects of CPDP-N on Antioxidant Enzyme Activities in H. pylori-Infected GES-1 Cells

**Figure 4:**
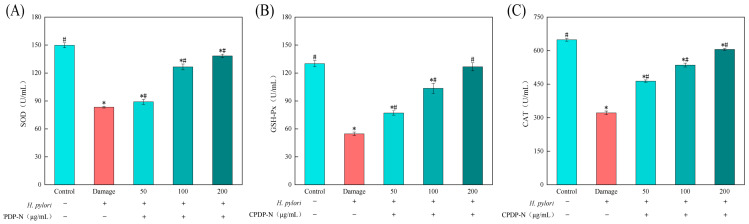
Effects of CPDP-N on antioxidant enzyme activities in *H. pylori*-infected GES-1 cells. (**A**) SOD activity; (**B**) GSH-Px activity; (**C**) CAT activity. * *p* < 0.05 vs. control; # *p* < 0.05 vs. *H. pylori*-infected group. In the figure, “+” indicates the addition of the corresponding substance, and “−” indicates that it has not been added. Data are presented as mean ± SD (*n* = 3).

SOD, CAT, and GSH-Px serve as the major line of enzymatic defense against intracellular oxidative damage [20]. Figure 4 illustrates that, relative to the control, the activities of SOD, CAT, and GSH-Px in the *H. pylori*-infected group declined markedly (*p* < 0.05) to approximately 55.65%, 42.04%, and 49.58% of control levels, respectively. This marked suppression of antioxidant enzyme activities indicates that *H. pylori* infection severely compromised the endogenous antioxidant defense capacity of GES-1 cells, which likely contributed to the excessive ROS accumulation observed in Section 3.2. CPDP-N pretreatment dose-dependently elevated the activities of all three enzymes. At 200 μg/mL CPDP-N, GSH-Px activity returned to a level comparable to that of the control group (*p* > 0.05), while SOD and CAT activities approached control values, indicating that CPDP-N effectively preserved the cellular antioxidant defense system against *H. pylori*-induced impairment.

Under normal physiological conditions, intracellular ROS levels are maintained within a narrow range by the coordinated action of antioxidant enzymes [18]. The observed decreases in SOD, CAT, and GSH-Px activities impair the cellular capacity to scavenge ROS, thereby intensifying oxidative stress and driving a self-reinforcing cycle of injury. In this vicious cycle, ROS overproduction overwhelms and inactivates the very enzymes responsible for their clearance. In the present study, CPDP-N pretreatment effectively broke this cycle by restoring antioxidant enzyme activities toward control levels, thereby facilitating ROS clearance and protecting GES-1 cells from oxidative damage.

### 3.4. CPDP-N Alleviated H. pylori-Induced Cell Cycle Arrest in GES-1 Cells

Under normal conditions, cell proliferation and apoptosis are maintained in a dynamic equilibrium. Following *H. pylori* infection of GES-1 cells, the virulence factors released can trigger inflammatory responses and disrupt cell cycle progression, ultimately leading to apoptosis [21,22]. Therefore, effectively alleviating the cell cycle arrest induced by *H. pylori* infection is critical for attenuating GES-1 cell injury.

**Figure 5:**
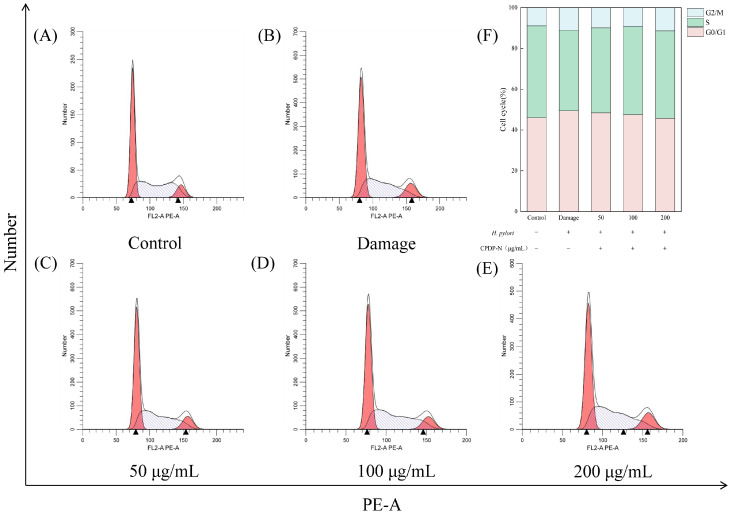
Effect of CPDP-N on the cell cycle in *H. pylori*-infected GES-1 cells. (**A**–**E**) Representative flow cytometry histograms; (**F**) Quantification of cell cycle phase distribution. In the figure, “+” indicates the addition of the corresponding substance, and “−” indicates that it has not been added. Data are presented as mean ± SD.

Figure 5 shows that the proportion of cells in the G0/G1 phase rose to 49.40% in the *H. pylori*-infected group relative to the control. This finding indicates that *H. pylori* infection induced G0/G1 phase arrest in GES-1 cells, which blocked entry into the S phase, halted DNA synthesis, and ultimately triggered apoptosis. This finding is consistent with Kang et al. [23], who reported that *H. pylori* infection perturbs the cell cycle and induces apoptosis. After pretreatment with rising concentrations of CPDP-N, the G0/G1 population progressively declined, whereas the S phase population increased, indicating that cells were able to re-enter DNA synthesis. These results demonstrate that CPDP-N pretreatment effectively alleviated the *H. pylori*-induced cell cycle arrest.

### 3.5. CPDP-N Improves Mitochondrial Membrane Potential Dissipation Induced by H. pylori Infection

Mitochondria are essential organelles that play critical roles not only in energy production but also in the regulation of apoptosis. During oxidative phosphorylation, electron transfer reactions on the inner mitochondrial membrane drive protons from the mitochondrial matrix into the intermembrane space, establishing a proton electrochemical gradient that generates the mitochondrial membrane potential. Upon *H. pylori* infection, released cytotoxins induce excessive ROS production, which triggers mitochondrial stress and dysfunction, ultimately leading to mitochondrial membrane potential dissipation and cell death [24].

**Figure 6:**
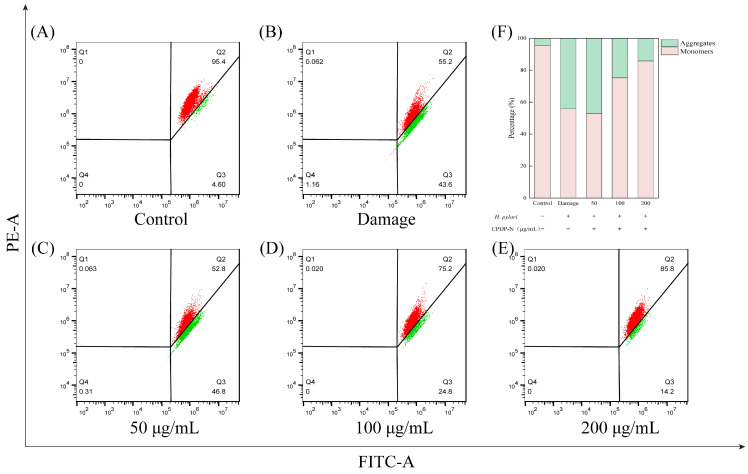
Mitochondrial membrane potential in *H. pylori*-infected GES-1 cells following CPDP-N treatment. (**A**–**E**) Representative flow cytometry histograms; (**F**) Red and green fluorescence distribution of mitochondrial membrane potential in each group. In the figure, “+” indicates the addition of the corresponding substance, and “−” indicates that it has not been added. Data are presented as mean ± SD.

As shown in Figure 6, in the blank control group, the cell population was predominantly localized in the upper right quadrant with strong red fluorescence, and the mitochondrial membrane potential was 95.4%. The *H. pylori*-infected damage group exhibited marked dissipation of mitochondrial membrane potential, evidenced by a downward shift in the cell population and a significant decline in red fluorescence intensity, with the membrane potential dropping to 55.20%. These results indicate that *H. pylori* infection caused severe mitochondrial damage in GES-1 cells, with a substantial amount of JC-1 remaining in the cytosol in monomeric form. The VacA toxin is one of the major effector molecules through which *H. pylori* disrupts mitochondrial function. Excessive ROS generated during infection attack cardiolipin and iron–sulfur clusters in respiratory chain complexes, establishing a self-amplifying loop between membrane potential decline and ROS overproduction. The greater the collapse of membrane potential, the more severe the electron leakage, the higher the ROS production, and the further the aggravation of mitochondrial damage.

CPDP-N treatment at all tested concentrations significantly elevated the mitochondrial membrane potential. At 200 μg/mL CPDP-N, the membrane potential recovered to 85.80%, indicating that CPDP-N effectively alleviated *H. pylori*-induced mitochondrial damage. Ren et al. demonstrated that antioxidant peptides derived from broken rice increased mitochondrial membrane potential and enhanced cell viability by reducing intracellular ROS levels, an effect attributable to their antioxidant activity [25]. Intervention with antioxidant substances can mitigate cellular oxidative stress and thereby restore mitochondrial membrane potential. Two possible causal pathways may underlie the relationship between CPDP-N-mediated ROS reduction and membrane potential recovery: (i) the peptides directly scavenge free radicals, attenuating oxidative damage to respiratory chain complexes and preserving proton pump function; or (ii) the peptides may activate the endogenous antioxidant system, strengthening the cell’s intrinsic defense capacity, which is a mode of indirect protection that is often more durable than simple exogenous scavenging. Corn protein-derived bioactive peptides inherently possess such properties [18], which may be a key factor contributing to the pronounced efficacy of CPDP-N.

### 3.6. CPDP-N Attenuates Apoptosis in H. pylori-Infected GES-1 Cells

Apoptosis plays a critical role in gastric injury. Therefore, inhibiting apoptosis in GES-1 cells may prevent the progression of *H. pylori*-induced gastric damage. Attenuating pro-apoptotic signals while enhancing anti-apoptotic signals represents a key strategy for suppressing apoptosis [25]. Bax and Bad promote the apoptotic process, whereas Bcl-2 and Bcl-xl counteract it.

As shown in Table 2, the *H. pylori*-infected group exhibited significantly elevated mRNA expression of *Bax* and *Bad* (*p* < 0.05) and significantly reduced expression of *Bcl-2* and *Bcl-xl* (*p* < 0.05). Overexpression of pro-apoptotic factors accelerates apoptosis, indicating that *H. pylori* infection promotes apoptotic cell death in GES-1 cells, consistent with the findings of Hsu et al. [26]. The *H. pylori* virulence factor VacA is internalized and translocates to the mitochondria, where it triggers mitochondrial fragmentation and the release of cytochrome C and other apoptogenic factors, thereby initiating apoptosis. Moreover, excessive ROS production is recognized as a major driver of apoptosis [27,28]. Taken together with the findings in Section 3.2, Section 3.3, Section 3.4 and Section 3.5, *H. pylori* infection likely triggers mitochondrial impairment and oxidative stress via excessive ROS production, resulting in cell cycle arrest and accelerated apoptosis in GES-1 cells. CPDP-N pretreatment at all tested concentrations significantly downregulated the expression of *Bax* and *Bad* (*p* < 0.05) and upregulated that of *Bcl-2* and *Bcl-xl* (*p* < 0.05). These results demonstrate that CPDP-N markedly attenuates apoptosis, suggesting that its preventive effect against *H. pylori*-induced gastric cell injury is closely associated with the inhibition of apoptotic cell death.

Corn protein hydrolysates have been reported to inhibit the expression of apoptosis-related proteins and mRNAs, including *caspase-3* and *Bax*, and this anti-apoptotic effect has been linked to their antioxidant activity [29]. Wang et al. [30] reported that corn glycopeptides regulated the expression of apoptosis-related proteins through their potent antioxidant activity, thereby antagonizing ethanol-induced hepatocyte apoptosis. Ma et al. [31] found that corn protein hydrolysates inhibited alcohol-induced hepatocyte apoptosis by upregulating anti-apoptotic gene expression and preserving mitochondrial membrane integrity, which is consistent with the findings of the present study. Taken together, these results suggest that CPDP-N may counteract *H. pylori*-induced apoptosis by enhancing cellular antioxidant capacity, accelerating ROS clearance, reducing mitochondrial damage, and alleviating oxidative stress. The present study was conducted exclusively in the GES-1 cell line. While GES-1 cells are a well-established model for studying *H. pylori*–gastric epithelial interactions, they are SV40-immortalized and may not fully recapitulate the behavior of normal gastric epithelial cells, particularly with respect to cell cycle regulation and apoptosis. Validation of the key findings in a non-transformed system, such as primary gastric epithelial cells or gastric organoids, will be necessary in future studies.

### 3.7. Effects of CPDP-N on Pro-Inflammatory Cytokine Levels in H. pylori-Infected GES-1 Cells

**Figure 7:**
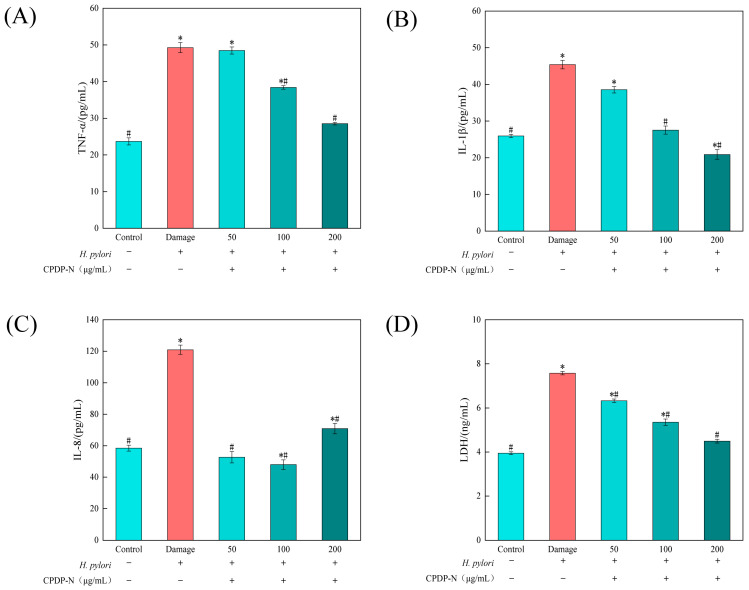
Effects of CPDP-N on pro-inflammatory cytokine levels and LDH release in *H. pylori*-infected GES-1 cells. (**A**) Intracellular TNF-α levels; (**B**) Intracellular IL-1β levels; (**C**) Intracellular IL-8 levels; (**D**) LDH levels. * *p* < 0.05 vs. control; # *p* < 0.05 vs. *H. pylori*-infected group. In the figure, “+” indicates the addition of the corresponding substance, and “−” indicates that it has not been added. Data are presented as mean ± SD (*n* = 3).

*H. pylori* infection of GES-1 cells induces oxidative stress and inflammatory responses. Overexpression of pro-inflammatory cytokines is a major driver of inflammation, and their intracellular levels correlate positively with the severity of the inflammatory response [32]. As shown in Figure 7, compared with the control group, the *H. pylori*-infected group exhibited significantly increased intracellular levels of TNF-α, IL-1β, and IL-8, as well as elevated LDH (*p* < 0.05), with increases of 107.80%, 75.13%, 106.81%, and 92.39%, respectively. This observation agrees with the report by Huang et al., which demonstrated that *H. pylori* infection increases pro-inflammatory cytokine levels in GES-1 cells [33].

Overexpression of TNF-α can induce the release of other pro-inflammatory cytokines such as IL-1β and IL-8, leading to cellular injury and apoptosis; therefore, it is considered a major driver of inflammation [34]. As shown in Figure 7A, *H. pylori* infection significantly increased intracellular TNF-α levels (*p* < 0.05), indicating a pronounced inflammatory response. Compared with the *H. pylori*-infected group, all CPDP-N-treated groups exhibited significantly reduced intracellular TNF-α levels in a dose-dependent manner (*p* < 0.05). At 200 μg/mL CPDP-N, the TNF-α level was significantly decreased by 42.09% and did not differ significantly from that of the control group (*p* > 0.05), demonstrating that CPDP-N pretreatment suppressed TNF-α release and attenuated the inflammatory response.

Increased levels of pro-inflammatory cytokines serve as direct triggers of the inflammatory cascade. *H. pylori* infection is known to upregulate pro-inflammatory cytokines, promoting neutrophil infiltration and eliciting a robust inflammatory response in host cells. Among them, IL-8 and IL-1β are pivotal mediators in *H. pylori*-related pathologies and are closely associated with the onset of gastritis and gastric ulcers [35]. As shown in Figure 7B,C, at a CPDP-N concentration of 100 μg/mL, the IL-1β level decreased by 39.37% and was not significantly different from that of the control group (*p* > 0.05). Notably, at a CPDP-N dose as low as 50 μg/mL, the intracellular IL-8 level was already significantly reduced to a level comparable to that of the control group, demonstrating the potent efficacy of CPDP-N in lowering IL-8. Studies have shown that IL-8 is the most markedly elevated pro-inflammatory cytokine following *H. pylori* infection, and persistently high IL-8 levels represent an important indicator of gastric cell carcinogenesis. CPDP-N intake may therefore significantly prevent the elevation of IL-8, reducing the risk of inflammation and malignant transformation. Li et al. [28] demonstrated that *H. pylori* infection markedly elevated intracellular IL-8 levels in GES-1 cells, whereas tetramethylpyrazine pretreatment significantly attenuated both IL-8 protein production and mRNA expression.

LDH is an intracellular enzyme that is stably retained within the cell under normal conditions. Upon *H. pylori* infection, virulence factors disrupt the cell membrane, causing LDH leakage and a significant increase in LDH levels in the culture medium; thus, LDH serves as a classic indicator of cellular damage. Measuring LDH in the culture medium therefore directly reflects cell membrane integrity. Figure 7D shows that CPDP-N treatment markedly suppressed LDH release in a dose-dependent fashion by 16.50%, 29.36%, and 40.73%, respectively (*p* < 0.05). At 200 μg/mL CPDP-N, no significant difference in LDH release was observed between the CPDP-N-treated and control groups (*p* > 0.05), indicating that CPDP-N pretreatment counteracted *H. pylori*-induced membrane damage, preserved cell membrane integrity, and reduced LDH leakage.

CPDP-N pretreatment may mitigate the inflammatory response through multiple mechanisms. First, it exerts an anti-adhesive effect against *H. pylori*, reducing the bacterial load adhering to the surface of GES-1 cells and consequently decreasing the release of virulence factors, thereby attenuating the inflammatory response. Second, the potent antioxidant activity of CPDP-N likely represents another important contributor, as it can enhance the overall cellular antioxidant capacity to combat *H. pylori*-induced inflammation. Furthermore, CPDP-N is rich in amino acids such as Ala, Glu, Gln, Phe, and Pro, which can serve as carbon skeletons for metabolic intermediates, promoting the tricarboxylic acid (TCA) cycle, accelerating cellular metabolism, and regulating the production of intracellular antioxidant enzymes. This may lead to a reduction in TNF-α release and subsequently lower IL-1β and IL-8 levels, collectively alleviating the inflammatory response. Taken together, these findings indicate that CPDP-N pretreatment alleviates *H. pylori*-induced inflammatory injury in GES-1 cells through multiple synergistic pathways.

### 3.8. CPDP-N Upregulates IL-10 in H. pylori-Infected GES-1 Cells

**Figure 8:**
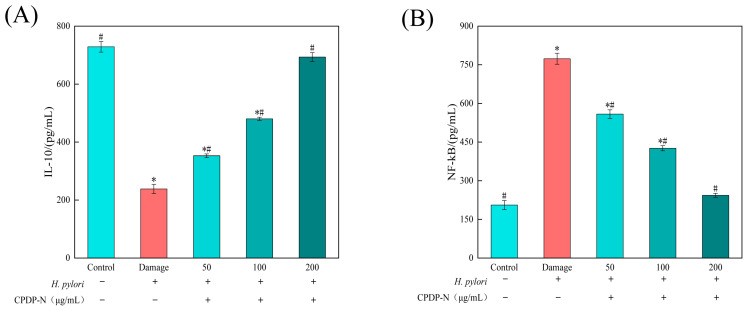
Modulation of IL-10 and NF-κB p65 by CPDP-N in *H. pylori*-infected GES-1 cells. (**A**) Intracellular IL-10 levels; (**B**) Intracellular NF-κB p65 levels. * *p* < 0.05 vs. control; # *p* < 0.05 vs. *H. pylori*-infected group. In the figure, “+” indicates the addition of the corresponding substance, and “−” indicates that it has not been added. Data are presented as mean ± SD (*n* = 3).

IL-10 levels in the *H. pylori*-infected group declined significantly by 67.27% compared with the control (*p* < 0.05, Figure 8A), further confirming that infection aggravated the inflammatory response, in agreement with the findings in Section 3.7. CPDP-N treatment at all doses significantly increased intracellular IL-10 levels (*p* < 0.05) by 47.92%, 101.39%, and 190.72% in a dose-dependent manner. At 200 μg/mL, IL-10 levels were restored to a level comparable to that of the control group (*p* > 0.05), indicating that CPDP-N effectively strengthened the anti-inflammatory response. IL-10 has been reported to suppress NF-κB activity and its binding to DNA, which in turn reduces TNF-α secretion and alleviates the inflammatory response [36,37]. Collectively, CPDP-N may protect against cellular injury by increasing intracellular IL-10 levels and enhancing ROS scavenging capacity.

### 3.9. CPDP-N Reduces NF-κB p65 Protein Abundance in H. pylori-Infected GES-1 Cells

NF-κB, a central transcription factor, is deeply involved in orchestrating inflammatory responses and plays a pivotal role in immune regulation during bacterial infection [38]. Figure 8B shows that the NF-κB p65 level in the *H. pylori*-infected group rose significantly to 2.75-fold (*p* < 0.05), indicating that infection elevated NF-κB p65 protein abundance and triggered an inflammatory response. This finding aligns with a previous report by Tian et al., who demonstrated that *H. pylori* infection elevates NF-κB p65 levels in GES-1 cells [38]. CPDP-N pretreatment significantly reduced NF-κB p65 levels in a dose-dependent manner (*p* < 0.05). At 200 μg/mL, the intracellular NF-κB p65 level decreased to 243.58 pg/mL and was not significantly different from that of the control group (*p* > 0.05), demonstrating that CPDP-N intervention reduced NF-κB p65 protein abundance, alleviated the inflammatory response, and restored NF-κB p65 levels to normal. Tian et al. [39] found that lipopolysaccharide (LPS) produced by *H. pylori* can activate the NF-κB signaling pathway in gastric mucosal epithelial cells, thereby triggering an inflammatory response. The lowering of NF-κB p65 levels by CPDP-N may involve two complementary mechanisms. First, its anti-adhesive properties could reduce the bacterial load on GES-1 cells, thereby limiting LPS entry and subsequent TLR4 engagement, which in turn dampens downstream inflammatory signaling. Second, CPDP-N may directly suppress the production of pro-inflammatory cytokines, thereby attenuating NF-κB activation and mitigating the inflammatory cascade.

**Figure 9:**
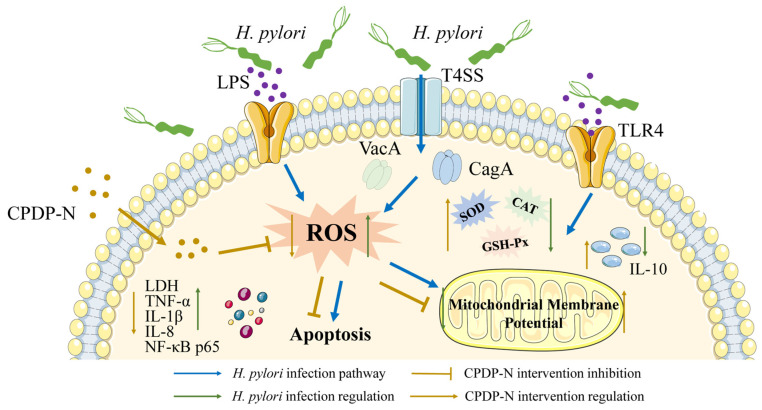
Proposed protective mechanism of CPDP-N against *H. pylori* infection-induced injury.

The present data directly demonstrate that CPDP-N pretreatment reduces intracellular ROS levels, restores mitochondrial membrane potential, alleviates cell cycle arrest, normalizes apoptosis-related gene expression, decreases pro-inflammatory cytokine levels (TNF-α, IL-1β, IL-8), elevates IL-10, and reduces NF-κB p65 protein abundance in *H. pylori*-infected GES-1 cells (Figure 9). While the effective concentrations tested in vitro (50–200 μg/mL) are within the range commonly employed for peptide bioactivity studies, their physiological relevance cannot be determined from the present data. Pharmacokinetic studies in animals will be needed to establish whether orally administered CPDP-N can reach gastric tissue concentrations comparable to those tested in this study.

Regarding the mechanistic readouts, NF-κB p65 was assessed by ELISA detecting total p65 protein, an approach that does not distinguish between changes in protein abundance and phosphorylation-dependent activation or nuclear translocation. Consequently, the observed reduction in NF-κB p65 levels is consistent with, but does not directly demonstrate, modulation of NF-κB signaling, and confirmatory evidence from phospho-p65 or phospho-IκBα immunoblotting, or pathway-specific inhibitor experiments, is still needed. Furthermore, a CPDP-N-only control group was included only in the viability assay; its absence in the mechanistic experiments means that potential basal effects of the peptide mixture on oxidative stress, cell cycle progression, mitochondrial function, apoptosis-related gene expression, and inflammatory mediators cannot be fully separated from its protective effects against *H. pylori* infection.

## 4. Conclusions

The present study demonstrates that CPDP-N protective effects are associated with reduced intracellular ROS levels, preserved mitochondrial membrane potential, normalized expression of apoptosis-related genes, decreased pro-inflammatory cytokines, elevated IL-10, and lower NF-κB p65 protein levels. However, the proposed mechanisms remain to be confirmed through direct biochemical and pharmacological approaches. In vivo validation in animal models of *H. pylori* infection and, ultimately, clinical studies, will be necessary before CPDP-N can be considered a dietary intervention. The study was also limited to a pretreatment design, and whether CPDP-N can exert therapeutic benefits when administered during or after infection remains to be investigated. Addressing these methodological gaps will be an important step toward translating the current findings into practical applications.

## Figures and Tables

**Table 1:** Primers information for the qRT-PCR experiment.

Gene	Forward Sequence	Reverse Sequence
*GAPDH*	CCACATCGCTCAGACACCAT	GGCAACAATATCCACTTTACCAGAG
*Bax*	ATGGGCTGGACATTGG	GGGACATCAGTCGCTTCAG
*Bad*	CCAGAGTTTGAGCCGAGTGA	GATGATGCTTGCCGGAGCC
*Bcl-2*	CATGTGTGTGGAGAGCGTCAA	GCCGGTTCAGGTACTCAGTCA
*Bcl-xl*	TCCTTGTCTACGCTTTCCACG	GGTCGCATTGTGGCCTTT

**Table 2:** Effects of CPDP-N on cell apoptosis in *H. pylori*-infected GES-1 cells.

Group	*Bax*	*Bad*	*Bcl-2*	*Bcl-xl*
Control	1.00 ± 0.05 #	1.00 ± 0.16 #	1.00 ± 0.10 #	1.00 ± 0.07 #
*H. pylori*-infected damage	8.19 ± 0.29 *	1.49 ± 0.41 *	0.81 ± 0.09 *	0.11 ± 0.02 *
50 μg/mL	5.52 ± 0.34 *#	1.19 ± 0.03 *#	0.88 ± 0.16 *	0.43 ± 0.09 *#
100 μg/mL	1.79 ± 0.22 *#	0.91 ± 0.14 #	0.89 ± 0.10 *	0.70 ± 0.09 *#
200 μg/mL	0.96 ± 0.06 #	0.88 ± 0.22 *#	0.93 ± 0.26 #	0.86 ± 0.03 *#

Notes: * *p* < 0.05 vs. control; # *p* < 0.05 vs. *H. pylori*-infected group. Data are presented as mean ± SD (*n* = 3).

## Data Availability

The original contributions presented in this study are included in the article. Further inquiries can be directed to the corresponding authors.
